# ChatGPT and large language models in academia: opportunities and challenges

**DOI:** 10.1186/s13040-023-00339-9

**Published:** 2023-07-13

**Authors:** Jesse G. Meyer, Ryan J. Urbanowicz, Patrick C. N. Martin, Karen O’Connor, Ruowang Li, Pei-Chen Peng, Tiffani J. Bright, Nicholas Tatonetti, Kyoung Jae Won, Graciela Gonzalez-Hernandez, Jason H. Moore

**Affiliations:** 1 Department of Computational Biomedicine, Cedars Sinai Medical Center, Los Angeles California, USA; 2 Department of Biostatistics, Epidemiology, and Informatics, University of Pennsylvania, Philadelphia, Pennsylvania USA

## Abstract

The introduction of large language models (LLMs) that allow iterative “chat” in late 2022 is a paradigm shift that enables generation of text often indistinguishable from that written by humans. LLM-based chatbots have immense potential to improve academic work efficiency, but the ethical implications of their fair use and inherent bias must be considered. In this editorial, we discuss this technology from the academic’s perspective with regard to its limitations and utility for academic writing, education, and programming. We end with our stance with regard to using LLMs and chatbots in academia, which is summarized as (1) we must find ways to effectively use them, (2) their use does not constitute plagiarism (although they may produce plagiarized text), (3) we must quantify their bias, (4) users must be cautious of their poor accuracy, and (5) the future is bright for their application to research and as an academic tool.

## Introduction

Since the release of ChatGPT in November 2022 [1], academia has expressed divergent opinions about the use of this technology. This artificial intelligence (AI)-based chatbot interacts with users in a conversational way, using human-like language to answer questions and generate content. It is also trained to create computer code. ChatGPT tracks previous prompts and responses, correcting and adapting subsequent answers given the sequence of inputs and outputs. ChatGPT is powered by a LLM, a type of deep learning model that emerged around 2018 [2]. These models are trained on massive amounts of publicly available text data, such as books, articles, and webpages, to generate human-like responses in conversations.

Academia faces a technological evolution driven by ChatGPT and LLMs. On the one hand, the potential of ChatGPT and LLMs in education and research is exciting. It can be used as a classroom aid to provide quick answers to questions, or a learning tool that assists in literature reviews and article outlines. On the other hand, there are also unsettling ethical issues to be considered [3–5]. For example, LLMs may adopt bias, perpetuate stereotypes in the training dataset, and present false information as truth. In this article, we discuss the advantages and concerns surrounding this AI technology. We examine the use of LLMs like ChatGPT in education, programming, and academic writing. We also comment on bias, scalability and accessibility of AI models like ChatGPT. Our hope is that this article will generate interest and further discussion related to incorporating LLM-based chatbots in academia, while taking ethical issues under careful consideration. We close with a summary of our stance on use of LLM based chatbots in academia.

## Background

There are several points of background required to understand ChatGPT and other LLMs discussed in this editorial. First, a key advance of this technology is its iterative ability, where previous responses and outputs tune subsequent outputs. Second, there are two versions of ChatGPT: a free version using the model version 3.5, and the paid version that currently uses the model version 4.0. It is likely that OpenAI used the feedback from the free version to power improvements that made it into the paid version. An additional important feature of version 4.0 is that it can accept image inputs. For example, it can use a drawing of an idea for a website to produce the code required for building that website. There are also other platforms, for example from Google called Bard, and other LLMs such as BLOOM, an open access 176B parameter multilingual LLM that can be used to power chat-like interfaces and for deployment of other applications. Finally, an important concept is that of “prompt engineering” and acting as a “prompt engineer”, which is that due to the iterative nature of ChatGPT or Bard and the sensitivity to the exact choice of the text prompt, the ability to successfully induce desired outputs is not always trivial. Recently, a job posting for a prompt engineer was widely circulated on the internet with vague required qualifications offering up to > $300,000 per year in salary, highlighting the hype around LLMs used for chat.

A fundamental challenge with LLM-based chatbots is that they can present false information as truth, also known as “hallucination”. In fact, ChatGPT and Bard can misrepresent their capabilities. For example, when asked if it could help find relevant publications to cite in a review paper, ChatGPT confirmed that it could, but then proceeded to make up a list of five entirely fictional publications. On repeat trials, sometimes it listed one or two real papers, but other times, a paper was made up or the paper was real but had nothing to do with the inquiry, other than perhaps having the same author. Thus, an answer generated by a large language model can be formatted correctly but not necessarily be factual. One needs to remember that it is not a human: it's not trained to respond in a way that accurately reflects its own capabilities and limitations, but rather, it is trained to construct textual utterances modeled after similar ones found in its training text given the prompt. These issues limit the utility of LLM chatbots in some domains, such as the medical field, where facts are essential to ensure the best health outcomes. For example, it would not be wise to base treatment plans on outputs from ChatGPT, at least in its current state, because this would lead to a large proportion of misdiagnoses and ineffective treatments that could cause patient harm. Because of this limitation, any usage of LLMs should carefully scrutinize the outputs for accuracy.

## ChatGPT for academic writing

### Ethics and rules of using text from ChatGPT

LLMs can benefit scientific research and writing, but how should their use be documented? The use of text generated by ChatGPT and other LLMs is an ethical gray area. Presenting work from someone else as one’s own work is considered plagiarism, but what if that text was generated by an AI model? Further, what if that text was generated through an iterative process where the author guided the AI? When it comes to AI-generated text, it’s not so clear where individual contribution ends and plagiarism begins. There are cases where identifying the source of writing is required, such as manuscript authorship. Indeed, we have already seen manuscripts where ChatGPT has been listed as author [6–8]. In one case, ChatGPT was listed as an author in the preprint [9] but missing as an author in the final publication [4]. Several journals have issued guidance about whether ChatGPT can be an author and what text is allowed from LLM in the final publication. *Science* has declared that no AI generated text or figures are allowed in their published papers, and ChatGPT cannot be named as an author in their journal [10]. *Nature* issued similar guidance, stating that: (1) no LLM tool will be accepted as an author on a research paper, and (2) researchers using LLM tools in their research should document how they were used in the methods and/or acknowledgements section [11].

### ChatGPT as a writing and editing tool

According to the 2020 NSF Science and Engineering Indicator, 49% of the postdoctoral fellows trained in the United States were born overseas. In the fields of engineering, math, and computer science, 60% of PhD were awarded to international students [12]. Effective academic writing plays an essential role in the success and quality of academic publications, nevertheless, it is one of the major challenges facing international postdocs and students.

LMMs can provide benefits to researchers from all backgrounds, but particularly for non-native English speakers, as a writing and editing tool to enhance the quality of academic writing. Software-based editing tools have undergone significant advancements over time. From basic spelling checks implemented by document editors (e.g. Microsoft Word) to commercially available online services such as Grammarly, Scribbr, and Quillbot, these software-based editing tools are packed with various features to correct grammatical errors and improve writing clarity. However, these tools typically have a pre-defined set of evaluations of the writing sample and present a corresponding report based on these evaluations. In contrast, LMMs offer more flexibility in evaluations through the use of custom prompts. For instance, users can use a prompt such as “Can you explain what grammatical mistakes you have detected?” to request LMMs to provide explanations for the identified grammatical errors. The explanations offer users additional information to assess the accuracy of the report. Examples of designing creative prompts to improve grammar, readability, tone, intent, and others have been reported [13].

It is important to highlight that using LMMs as an editing tool differs from prompt-based text generation. As an editing tool, LMMs utilize texts authored by humans as a basis to make modifications and recommendations. This process is similar to asking a friend or colleague to proof-read a writing sample and offer suggestions. Consequently, employing LMMs with pre-written text is unlikely to raise ethical concerns such as plagiarism, which may arise when using prompt-based text generation by LMMs.

### How has ChatGPT been used to help write papers?

In some examples, manuscripts report the performance of ChatGPT on some test or task [4]. In another example, a response from ChatGPT was used to speculate on why taking Rapamycin may be beneficial using a specific philosophical argument [7]. In response to a short prompt, ChatGPT presented a logical introduction to Pascal’s Wager and Rapamycin along with a balanced discussion of how the argument may be applied to taking the drug. In a third example, a researcher presents the results of a conversation with ChatGPT around AI, chatbots using LLMs, and plagiarism in higher education [8]. In this example, ChatGPT did a good job summarizing ChatGPT, plagiarism, how ChatGPT might be used for plagiarism, and also how college professors might incorporate different types of assessments such as presentations and activities to avoid relying solely on potentially plagiarized essays for grades.

There are some ways researchers might want to avoid using LLMs. For example, researchers may be tempted to use ChatGPT to summarize fields to gain an overview, however, because ChatGPT will often present false information as truth, this is not advisable. It would be better to use resources like Wikipedia to get a broad overview of a topic. There are, however, new examples of LLMs developed specifically for providing accurate scientific information, such as perplexity.ai. In a prompt asking about our recent work, this tool found our preprint and put it in context of other related manuscripts with relevant, but not comprehensive, citations.

### ChatGPT and LLMs for grant proposal writing

The use of LLMs in grant proposals may require different rules, where already writing is commonly produced by staff helping the principal investigator (PI) with a proposal; for example, often postdoctoral fellows or graduate students will help a PI write a grant proposal related to their project that is later submitted by a PI. It is also common to use professional writing staff or freelance grant proposal writers that, in some cases, produce the entire text of a proposal submitted by a PI. Working with staff and even contractors to draft proposals is not against NIH policy. Although a proposal is associated with a PI, it is formally submitted by the institution, and therefore a sole author is not assumed. Depending on the trends in LLM usage for grants and papers, federal funding agencies may need to issue guidance on what is and is not allowed.

ChatGPT has undoubtedly already been applied to help write grant proposals. In one author's experience experimenting with how ChatGPT might help write grants, ChatGPT can help provide some ideas for interesting directions, even suggesting sound logical reasons that we need to do research that could find their way into the significance section of NIH grants. ChatGPT can even provide general text that might be logically used in parts of the grant, such as the aims page. It can suggest specific experiments that might test a hypothesis of interest. A surprising effect for one author of this editorial was that it helped overcome writer's block in that simply having some text on the page was sufficient to move forward with editing. As Jodi Picoult said: “You can always edit a bad page. You can't edit a blank page.” However, grants must convey many complex ideas in a short amount of space, and as such, since much of the text from ChatGPT is so general and often repetitive, in our experience none of the text produced by ChatGPT made it into the final grant proposal.

### The future of LLMs: as a grant proposal reviewer?

Every year, thousands of grant proposals are reviewed by scientists, costing thousands of hours of effort and travel time. Despite this massive investment, the current review system is subjective; for example, some proposals sent to one study section are scored very poorly, while nearly the same proposal at another study section is funded. One could imagine that LLMs could eventually be used to create less subjective reviews, but there is a long road ahead and many layers of intelligence needed. This would enable faster review times and less wasted human time, which could then be devoted to more research. In anticipation that LLMs may be used for grant proposal review by individual reviewers, on June 23rd 2023 the NIH issued guidance in NOT-OD-23–149 that “the NIH prohibits NIH scientific peer reviewers from using natural language processors, large language models, or other generative Artificial Intelligence (AI) technologies for analyzing and formulating peer review critiques for grant applications and R&D contract proposals.” It seems unlikely that use of LLMs for grant proposal review will be embraced in the near future.

## Scalability and accessibility of LLMs

ChatGPT is not a one-of-a-kind tool and Large Language models (LLMs) have been around for a few years with varying abilities to generate meaningful and useful text. However, many of these tools are freely available as command line interfaces (CLIs) which might explain the quick rise to fame of ChatGPT. OpenAI cleverly engineered the GPT-3 model to be deployed at scale alongside an intuitive user interface. Anyone with an email address could start generating text. Public interest soared and ChatGPT gained 100 million active users in 2 months, a feat not even rivaled by the likes of Instagram, Facebook or TikTok.

With an estimated 13 million daily queries, ChatGPT occasionally struggled to meet the demand and queries could not be processed. As a comparison, Google search receives an estimated 8.5 billion daily queries. Deploying AI models at scale is a challenge that future AI technologies will have to face even for tech giants such as Google (Bard power by LaMDA) and Microsoft (AI powered Bing) that have announced AI integration within their respective search engines.

The computational hardware used to train AI models is costly both in terms of manufacturing and energy consumption during training and deployment. Speculation on the compute power OpenAI had access to (10,000 Nvidia V100s) puts the cost of the hardware close to $5 million dollars without factoring in research, development, and energy usage. A recent study estimated that the training of BERT (a 6 billion parameter LLM) would produce between 21 to 78 metric tons of CO_2_ to train to completion [14]. As a reminder, ChatGPT is a 175 billion parameter LLM and this only considers the cost of training and not any cost related to generating text in response to potentially billions of daily queries. Energetic and environmental strains related to large scale AI models could be reduced by preferring sustainable energy sources, increasing algorithm efficiency, and developing low-energy AI dedicated hardware.

OpenAI might have started off as a non-profit project but in the face of large-scale AI application costs there is an undeniable incentive to become financially profitable. ChatGPT offers a free tier as well as a $20/month premium subscription plan for preferential and efficient access to the model even during peak times. While AI has the potential to revolutionize education and academic research, paywalled access could further increase the divide between the wealthy and the poor. If AI services remain free, we must wonder what the hidden costs are and how will user information be used for profit—a common practice in free-to-use online services.

The question of data usages also shines a light on the ethical conundrum that has rattled the world of art with other generative AI models such as DALL-E and Mid-Journey: who owns the training data? Artists, writers, and musicians whose work constitutes a fundamental ingredient to the success of these models may find themselves slighted by the monetization of their work with no credit given.

## Education

ChatGPT holds promise across multiple levels of education, potentially serving a variety of supporting roles across K-12, undergraduate, and graduate education. A variety of commentaries have already emerged with respect to the use or potential for misuse of ChatGPT. Here we consider the potential role of ChatGPT from both the perspective of the student and educator.

LLMs provide benefits and opportunities in education for students by assisting with research and academic writing [15] or using them as an interactive study guide which may include generating practice exams and being provided immediate feedback [16, 17]. Opportunities for students at all levels include using ChatGPT as a source of creative inspiration, a way to get quick direct answers to specific questions, and a content generator (e.g. to draft, format, summarize, and/or edit). In higher education, LLM-based chatbots may be used to increase student engagement, facilitate group activities, create interactive learning tools, and provide immediate feedback and assessment [18–20].

The use of ChatGPT is not without challenges and limitations, both for learners and educators. Over reliance may hinder students ability to develop critical skills, such as writing [21]. Concerns have arisen regarding how students might easily use ChatGPT dishonestly, either to cheat in the completion of homework or exams, or to write reports/essays without citation in a manner that could be construed as plagiarism [22]. Data produced from ChatGPT may be inaccurate or biased [23], which users need to be aware of to ensure these deficiencies are not propagated in their works.

Despite these concerns, it should be argued that students can be taught to engage with ChatGPT in a constructive manner in line with the ethics or honor code of educational institutions. Ultimately the issue should not be ‘whether’ the student used ChatGPT, but ‘how’, not unlike the issue of how parents might help their children in the completion of assignments. For example, a student that turns in verbatim text generated by ChatGPT in response to a single request from that student to write that essay would be clearly undesirable. In contrast, a student who produces an essay by taking on the roles of prompt engineer, fact checker, and editor, should be viewed positively.

While the detection of cheaters remains an unsolved challenge for educators, if we start from the assumption that students are allowed, if not encouraged, to utilize ChatGPT, strategies could be adopted to account for this. For example students could be required to (1) submit their transcript of ChatGPT interaction, (2) contrast their final submission with the one generated by ChatGPT with tracked changes, or (3) point out errors made by ChatGPT, and how those errors were resolved in the students' submitted assignment. As asserted by CEO of OpenAI, Sam Altman: “Generative text is something we all need to adapt to. We adapted to calculators and changed what we tested for in math class, I imagine.” This is a more extreme version of that, no doubt, but also the benefits of it are more extreme, as well. Perhaps the arrival of ChatGPT just further highlights the numerable shortcomings in how our education system typically evaluates students.

A more important concern regarding students learning from a LLM is that it typically responds in a confident, and seemingly knowing manner, whether the provided information is accurate or not. Students should be made aware that the system is not as clever as it seems, and can unpredictably misrepresent or invent information. Ultimately, ChatGPT is not unlike using the internet for educational purposes, where most information should be digested with a degree of skepticism, requiring confirmation across multiple, ideally primary, sources.

Turning to educators, ChatGPT provides many new opportunities to facilitate course development and design, lesson planning [24], assessment [18], and evaluation [25]. As the use of ChatGPT and other LLMs by students is inevitable, educators will need to change the pedagogy and assessments in education, such as integrating the use of ChatGPT in lessons [26], and a focus more on the process rather than the final product for assessment. Teaching students the skills needed for the proper use of LLM-based chatbots, including the limitations of these tools, will be important to ensure students use them responsibly and disclose their use, as well as ensuring that all students have access to the tools so as not to create disparities in learning [17, 22].

## Assistance with programming

ChatGPT also promises to facilitate computer programming in terms of (1) learning to code or use specific packages, libraries, or frameworks, (2) writing new code, (3) interpreting existing code, (4) debugging existing code, (5) increasing the compactness or efficiency of code, or (6) translating code from one programming language to another. Upon inquiry, ChatGPT claims to be able to help with a wide variety of programming languages including Python, Java, JavaScript, C +  + , C, Ruby, Julia, PHP, SQL, HTML, CSS, and others.

As an educational programming resource, ChatGPT can be applied as an interactive instructor, answering questions about what language to use, code syntax and semantics, best practices, available libraries or packages, alternative approaches, integrated development environments (IDEs), and programming environments. ChatGPT can also generate simple/clear example code, complete with comments for each line, and a natural language summary of what the code does (highlighting the underlying function of key variables, methods, or packages included). In contrast to using google search, or sites like stackoverflow, geeksforgeeks, for ‘how-to’ coding questions, ChatGPT appears capable of directly providing the learner with a simple, interpretable, and relevant solution to a specific coding inquiry. Educators can also task ChatGPT with generating coding tutorials, as well as homework and exam questions related to specific programming topics.

For programmers of any skill level, ChatGPT can be tasked with automatically writing new code. Of note, there are already some examples of ChatGPT being directly integrated into IDEs to increase code writing productivity (e.g. respective plugins for JetBrains or Visual Studio), as well as other related AI applications for facilitating code writing (e.g. Github Copilot). However the utility and applicability of ChatGPT in writing code will ultimately rely on (1) the level of detail provided by the user in describing the function and parameters of the desired code and (2) on the scale and complexity of the requested code. Currently, ChatGPT is more likely to be successful in accurately writing smaller blocks of code, whereas its reliability in writing larger/more complex programs (e.g. a software package) is questionable.

Beyond writing new code, ChatGPT has the potential to serve a number of axillary functions for improving existing code. Code sharing on platforms like GitHub offer tremendous opportunities to accelerate research and development in computing. However, it can be a struggle to interpret code written by others (e.g. students or even our own code from years past) since there are often many valid ways to program a specific task. This is particularly true when code is poorly documented with limited, inaccurate, or ambiguous comments. A user can ask ChatGPT what a line or chunk of code does, and it will attempt to break it down into individual pieces, explaining variables, commands, and steps, as well as including a general summary of what it thinks that code is doing. In a related task, ChatGPT can be asked to add or correct comments in code as a means of automating code documentation. ChatGPT can also facilitate code debugging, although, as is the case in manual coding, identifying bugs that impact expected code function or performance are likely to be much harder to identify than those that simply prevent the code from running successfully. Furthermore, users can ask it to try and simplify existing code in an attempt to make it more compact, interpretable, or computationally efficient, or to translate code from one programming language to another.

In line with warnings for general use, ChatGPT may somewhat unpredictably present incorrect code as being correct. Further, it may be unaware or unable to anticipate edge cases that might break the code’s functionality under special circumstances, and it may not present the best or most efficient coding solution by default. Ultimately, code written by ChatGPT may offer a useful starting point, however both the code and comments that it generates should be checked and validated by both the programmer and users to confirm that it fully satisfies the intended purpose. It would be ill-advised to rely directly on code generated by ChatGPT for any high stakes applications, where security, liability, privacy, and trust are paramount. Thus it seems, at least for now, that experienced human programmers are far from obsolete.

## Bias

With the growth and proliferation of AI/ML solutions, there is increased scrutiny of algorithmic fairness, unintended harms, and equity related to the use of these solutions for marginalized groups. Unethically and irresponsibly designed AI/ML solutions deployed in the healthcare setting can exacerbate and perpetuate systematic biases and disparities for those from marginalized groups [27–29]. As companies race to innovate with LLMs like ChatGPT and others, encoded biases will be amplified, and harm will manifest. Until the root-causes of the encoded biases of LLMs are addressed, LLMs for clinical applications will suffer the same fate, propagating biases [30, 31]. The transformative work of addressing the root-causes of algorithmic bias starts with asking fundamental questions at the project design such as, is there a need for an AI solution and if so, for which purpose; what are the bias mitigation strategies; how will data exploitation be avoided; how interdisciplinary is the team (e.g., are there ethicists, legal, etc.)? Importantly, developing LLMs that reduce algorithmic bias and systemic racism requires action; technical approaches to assessing fairness must become part of the model evaluation process in a transparent manner, including disclosures for methods and metric selections. A multi-prong approach to mitigate bias in the data and model development pipeline could include Pre-processing algorithms such as Reweighing, Disparate Impact Remover, or Learning Fair Representations; In-Processing techniques such as Prejudice remove, Adversarial debiasing, or Discrimination aware; and Post-Processing such as Reject option classification or Equalized odds postprocessing [32]. When LLMs for clinical applications are socially, consciously, and transparently designed with such considerations, they can become an additional tool for promoting equity and improving access to care.

## Conclusion

To summarize our stance on the use of LLMs:

1. LLMs must be embraced.
  This editorial provides several examples and perspectives on how LLMs increase the efficiency of academic writing, education, and programming. Therefore, in our opinion, there is no question whether we should adopt these tools for all possible applications. LLMs must be embraced to increase efficiency of teaching and research across all disciplines.
2. Metrics quantifying LLM bias are required.
  The output of ChatGPT is from most publicly available text on the internet until 2021. The performance of ChatGPT and other LLMs therefore mimics the available text, and they are as biased as their training data. For example, ChatGPT is known to perpetuate stereotypes such as nurses being female and doctors being male, and this bias comes from the training data. It is difficult to define metrics that assess the level of bias in the training data and in the model outputs. When utilizing output from LLMs in studies of text analysis or generation, we must discuss inherent bias as a limitation.
3. Use of LLMs does not constitute plagiarism.
  Use of outputs from ChatGPT and LLMs may still seem like an ethical gray area in some senses. By design, LLMs take human text and ‘encode’ it for later use as a statistical model. Output from ChatGPT will match existing text on the internet, particularly if the sample is small enough. Thus, in a strict sense, LLMs like ChatGPT do produce plagiarism. However, ChatGPT does not do anything without a prompt. Lacking much prompt engineering and iteration, it feels more like plagiarism. After several rounds of prompt engineering to craft the output, it feels more like original work. Strictly, all work is crafted/engineered/prompted by the human in question. In that sense, the model output is that person's work. ChatGPT is just a tool like spellcheck or Grammarly. Like baking a cake from a box: the cake is the baker’s work, not the company that sells the cake mix. To be safe, we recommend any text generated by an LLM, or any human, be evaluated by plagiarism detection software for accidental close similarity to published text.
4. LLMs can generate false or inaccurate statements.
  In many contexts, LLMs have low accuracy. The model can generate correctly formatted information such as references, that do not exist. ChatGPT will misrepresent its knowledge; instead of responding “I don’t know”, it will readily provide fabricated information with apparent confidence. Users are responsible for citing the proper sources and ensuring the content is factual. Whatever is created by a LLM must be ‘adopted’ by the prompt engineer before sharing with others; thus, the output of a LLM belongs to the author of the prompt, and the author assumes responsibility for its validity and truthfulness.
5. The future.
  The future is bright for the application of LLMs to research and as an academic tool. While we have reviewed some of the current applications and discussion regarding LLMs, there are numerous creative applications to be developed and evaluated. Further, we see an opportunity to combine LLMs with other useful tools such as deep learning and automated machine learning. The future will unfold quickly and we will see many useful advances in the coming months. The challenge will be to keep up with the never ending open-source and commercial tools.
